# Omnidirectional zero-field ferromagnetic resonance driven by rotatable anisotropy in FeNi/FeMn bilayers without exchange bias

**DOI:** 10.1038/s41598-017-01639-x

**Published:** 2017-05-02

**Authors:** Wenfeng Wang, Guozhi Chai, Desheng Xue

**Affiliations:** Key Laboratory for Magnetism and Magnetic Materials of the Ministry of Education Lanzhou University, Lanzhou, 730000 People’s Republic of China

## Abstract

In this work, the zero- field GHz frequency permeability spectra and the rotatable anisotropy of FeMn/FeNi bilayers are investigated. Omnidirectional zero-field ferromagnetic resonance (FMR) driven by rotatable anisotropy can be achieved in the FeMn/FeNi bilayers without exchange bias. Instead of uniaxial anisotropy and exchange bias field, it is the rotatable anisotropy field that dominates in the unbiased bilayer, due to the absence of the exchange bias. The quantitative analysis of exchange bias field, uniaxial anisotropy and rotatable anisotropy is discussed by comparing the dynamical and static magnetic properties of FeNi single layer, biased and unbiased FeMn/FeNi bilayers.

## Introduction

Soft magnetic materials have been extensively utilized in many kinds of high frequency applications such as signal processing microwave devices^1–5^, high performance materials for radar absorbent^6^ *etc*. With the development of modern communication technologies, soft materials that possess excellent magnetic properties are urgently demanded, for instance, uniaxial anisotropy can meet the requirements. However, materials with uniaxial anisotropy have a critical drawback that restricts its usage in low-power-consumption and devices, for the in-plane orientation dependence of the magnetic films imposes restrictions on the application in magnetic devices. Hence, new types of films with in-plane isotropic magnetic properties driven by so-called rotatable anisotropy are developed, because of its application prospects in magnetic devices^7^. Rotatable anisotropy, and the magnetic thin films that possess such a property have been extensively investigated, since it was first found in the study of Permalloy thin films^8^. It has been proved that the rotatable anisotropy exists in many magnetic materials. For instance, in magnetic thin films with stripe domains, the rotatable anisotropy arises from the formation of rotatable stripe domains^9–12^. In amorphous magnetic thin films, rotatable anisotropy arises from the ripple effect^13–16^. In ferrite-doped CoFe films^17^, the rotatable anisotropy comes from the coupling between the doping-host of amorphous NiZn ferrite matrix with CoFe grains, epitaxial L1_0_ CoPt (111) thin films^18^. In exchange-biased antiferromagnetic (AFM)/ferromagnetic (FM) bilayers^19–25^, the rotatable anisotropy is explained by the exchange coupling at the interface of antiferromagnetic and ferromagnetic layers.

However, for all of the exchange biased systems mentioned above, the occurrence of rotatable anisotropy is always accompanied by an exchange bias field *H*_*e*_ (i.e. a shift of hysteresis loop along the axis of the applied field), and an enhancement of the coercivity. As a consequence, the magnetic materials that possess exchange bias effect would characterize unidirectional high frequency properties from which zero-field FMR frequency cannot be detected in some directions. In this paper, rotatable anisotropy in AFM/FM bilayer without exchange bias has been accomplished in which dynamic magnetic properties of the sample are dominated by rotatable anisotropy rather than unidirectional anisotropy as in biased films. The lack of exchange bias effect account for the in-plane omnidirectional permeability comparing to unbiased ferromagnetic-antiferromagnetic bilayer, it is of great importance for applications in micro devices.

## Results

**Figure 1:**
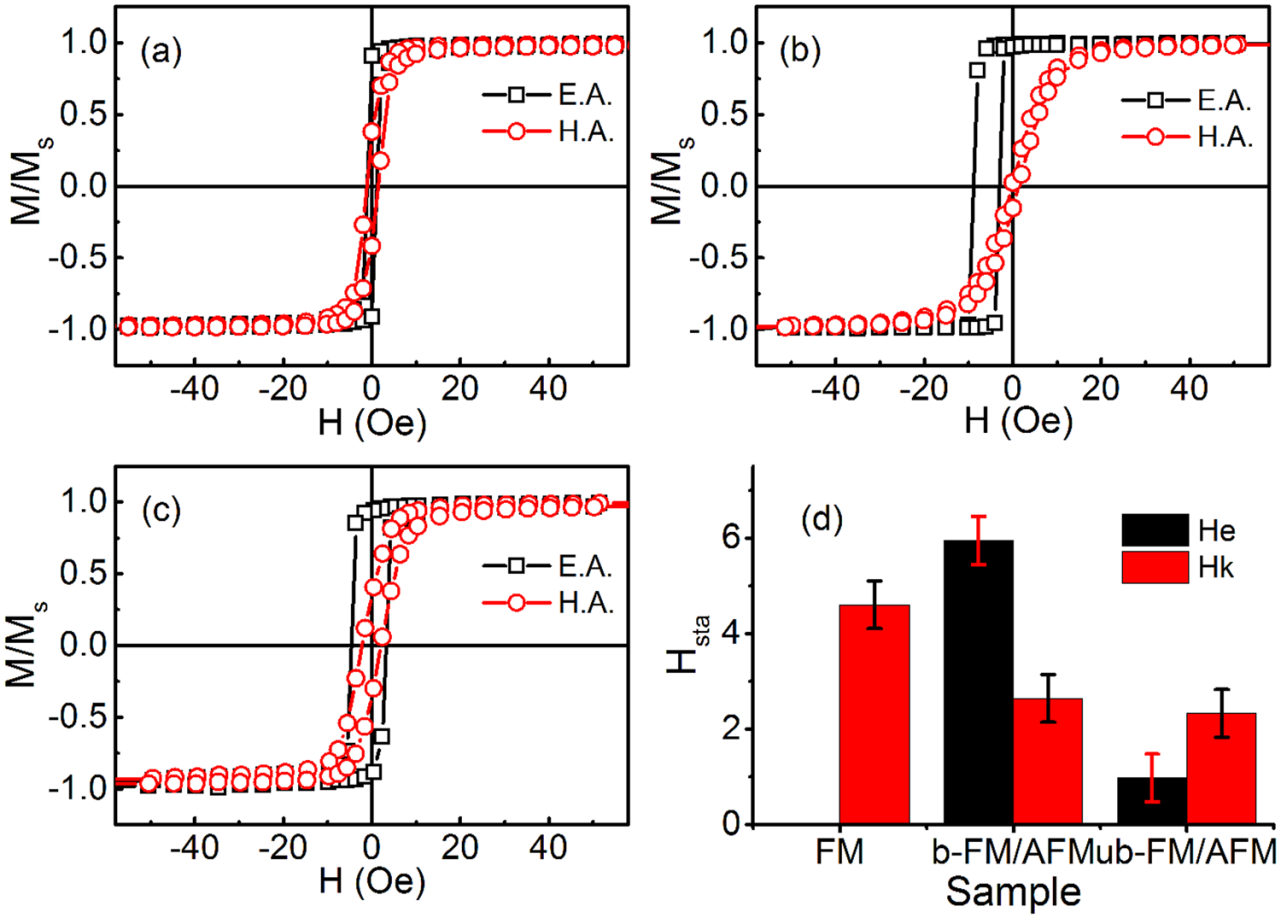
In-plane magnetic hysteresis loops and static anisotropies of the films. (**a**) Fe_20_Ni_80_ single layer with thickness of 50 nm, (**b**) biased Fe_20_Ni_80_/Fe_50_Mn_50_ bilayer (i.e. a permanent magnetic field was applied adjacent to the substrate during the depositing), (**c**) unbiased Fe_20_Ni_80_/Fe_50_Mn_50_ bilayer, measured along easy axis (E.A.) and hard (H.A.) axis, respectively. (**d**) the statistical result of static magnetic properties of the films, black shadow and red shadow are the exchange bias field ${H}_{{e}}^{{sta}}$ and uniaxial anisotropy field ${H}_{{k}}^{{sta}}$ extracted from the hysteresis loops respectively.

Figure 1 presents the representative easy and hard-axis magnetization hysteresis loops, measured with *H* parallel and perpendicular respectively to the easy axis (EA) of the films to illustrate the differences of (a) Fe_20_Ni_80_ single layer with thickness of 50 nm, (b) biased and (c) unbiased Fe_20_Ni_80_/Fe_50_Mn_50_ bilayer. From the shape changing of loops of all three samples as shown in Fig. 1(a–c), the remanence ratio (M_r_/M_s_) along hard axis (HA) of biased-AFM/FM is quite small. And the low remanence ratio may result in undetectable zero-field FMR when microwave magnetic field is applied parallel to the EA. On the contrary, a relative large remanence magnetization exists in the loops of unbiased-AFM/FM along HA as we can see from the comparison of Fig. 1(b,c). The omnidirectional zero-field FMR might be detected in this bilayer.

The values of anisotropy field, including uniaxial anisotropy field ${H}_{{k}}^{{sta}}$ and exchange bias field ${H}_{{e}}^{{sta}}$ (unidirectional anisotropy) can be determined by calculating the integral area of the loops along easy and hard axis of the reduced magnetizations as pointed out in the works of Neudert *et al*.^13^. In Fig. 1(a), the loop along the EA definitely center on the *H* = 0, which means the exchange bias field of Fe_20_Ni_80_ single layer is 0 because of the absence of antiferromagnetic layer. Thus, the film shows a typical uniaxial anisotropy with ${H}_{{k}}^{{sta}}$ = 4.4 Oe. Owing to the application of permanent magnetic field during sputtering, an exchange bias field ${H}_{{e}}^{{sta}}$, calculated using ${H}_{{e}}^{{sta}}$ = (*H*_*c*1_ + *H*_*c*2_)/2, of about 5.9 Oe appears in the biased layer as shown in Fig. 1(b), where *H*_*c*1_ and *H*_*c*2_ is coercivity of the increasing and decreasing part of the loops, respectively. The same consequences are reported in other works as well^21, 23^. Note that, in the results exhibited in Fig. 1(c), a slightly shift around 1 Oe of hysteresis loop of EA is observed in the unbiased bilayer, but much smaller than that of biased layer. That might be due to the exchange coupling between the FM spins and the small amounts of reversibly rotatable AFM spins in antiferromagnetic layer^26^. And a slightly enhancement of coercivity is observed in both biased and unbiased bilayers, which is consistent with others works^26, 27^, and can be explained as the enhanced pinning of the propagating domain wall in the ferromagnetic layer deriving from the interfacial magnetic frustration. The results of static uniaxial anisotropy ${H}_{{k}}^{{sta}}$ and exchange bias field ${H}_{{e}}^{{sta}}$ of all three samples extracted from hysteresis loops are plotted in Fig. 1(d) as comparison.

**Figure 2:**
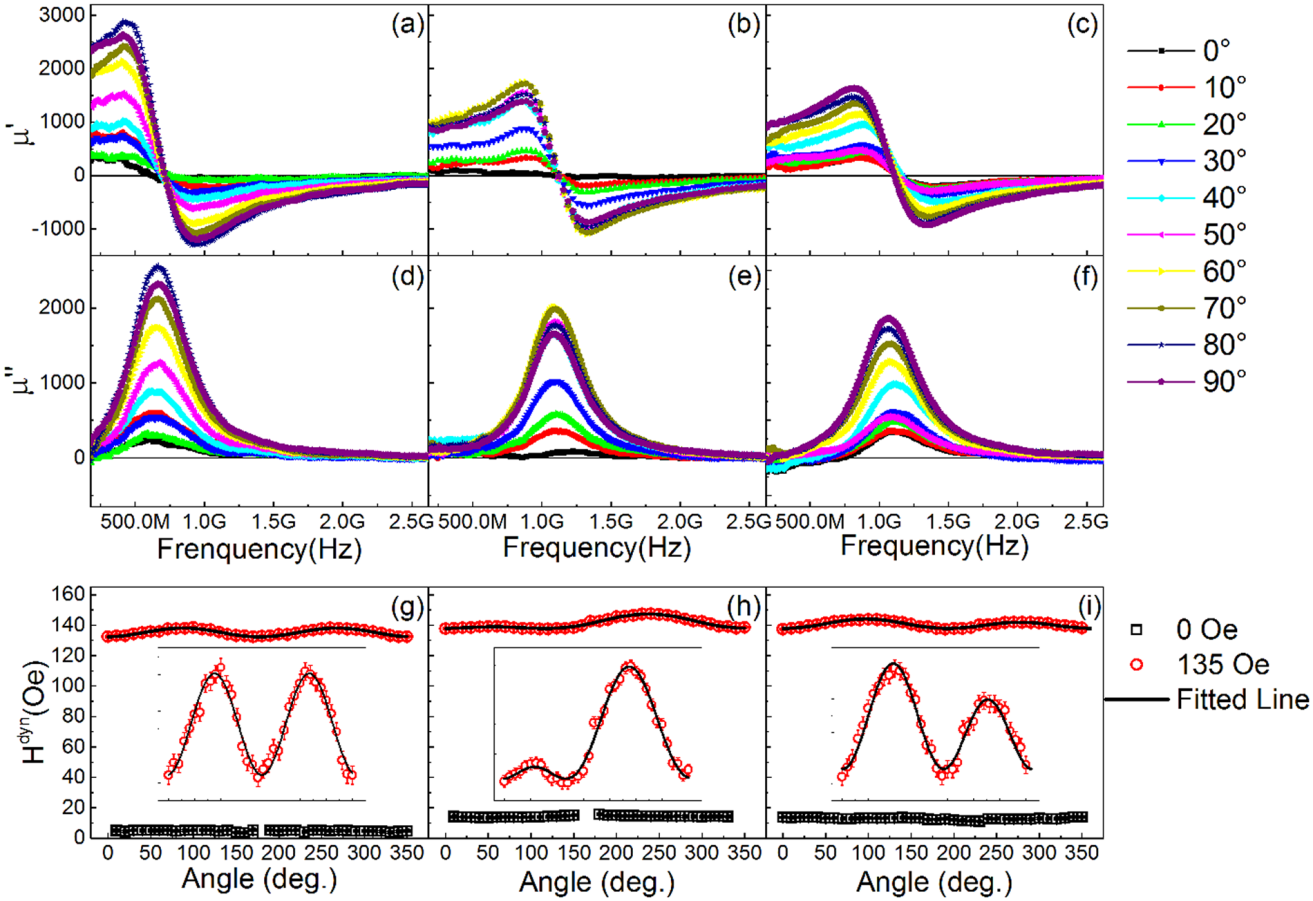
Dynamic properties of the FeNi single layer and AFM/FM bilayers. (**a**–**c**) zero-field real permeability of single FM layer, biased and unbiased AFM/FM bilayers, respectively, measured at angles from 0° to 90°. (**d**–**f**) are the zero-field imaginary permeability of corresponding films. (**g**–**i**) Dynamic anisotropy field *H* ^*dyn*^ fitted from the in-plane angle dependent frequencies of the FM layer, biased and unbiased AFM/FM bilayers, respectively, the black open squares are acquired from angle dependent resonance frequencies, the red circles are acquired from the resonance frequencies with a stationary magnetic field *H*_*0*_ = 135 Oe applied along the direction of the microwave, the black lines are fitted curves to the frequencies, and the magnifications are presented in terms of insert to show in detail.

As rotatable anisotropy cannot be obtained from regular static measurements of easy and hard hysteresis loops^9, 21^, an in-plane angle-dependent permeability spectrum was performed using vector network analyzer (VNA) with shorted micro-strip line, as presented in Fig. 2, aiming for analyzing the dynamic magnetic properties of the films. More details about the angle dependence measurement of permeability can be seen in refs 17 and 28. The first and second rows of Fig. 2 are zero-field real and imaginary permeability in GHz range of the FM single layer, biased AFM/FM and the unbiased AFM/FM films, respectively, measured at in-plane angle of 0° to 90°, the angle step is 10°. The *θ* shown in the figure is the angle between the microwave magnetic field and in-plane easy axis. Comparing to the FMR results of FM single layer as presented in Fig. 2(a,d), the resonance frequency *f*_*r*_ of the imaginary permeability of both biased AFM/FM [Fig. 2(b,e)] and unbiased AFM/FM [Fig. 2(c,f)] is evidently higher, i.e., an omnidirectional up-shift of the resonance frequency at the bilayers has been viewed, this can be understood as a consequence of rotatable anisotropy resulting from exchange coupling at the interface of FM and AFM layer^29–31^. Moreover, it is distinct that for single layer and biased bilayer, no visible resonant peak is found at some specific angles (e.g., 0°), nevertheless, the obvious resonances can be found under all angles for the unbiased bilayer, as we can see from comparison of real [Fig. 2(b,c,g)] and imaginary [Fig. 2(d–f)] permeability of the films.

## Discussion

To analyze the rotatable anisotropy of the films quantitatively, the dynamic anisotropy of the films *H* ^*dyn*^ are fitted from angle dependent resonance frequencies using an equation derived from Kittel’s Equation^32^

1 $${(\frac{2\pi f}{4\pi \gamma {M}_{s}})}^{2}={H}_{0}+{H}_{{rot}}^{{dyn}}+{H}_{{e}}^{{dyn}}\,\cos \,\theta +{H}_{k}^{dyn}\,\cos \,2\theta $$

where ${H}_{{e}}^{{dyn}}$, ${H}_{{k}}^{{dyn}}$ and *H*_0_ (135 Oe) are dynamic exchange bias field, dynamic uniaxial anisotropy field and applied static magnetic field. The fitting results are plotted in Fig. 2(g–i), for FM single layer, biased AFM/FM and unbiased AFM/FM, respectively, the black open squares represent the angle dependence of resonance frequencies of the films with *H*_0_ = 0 Oe. There are no resonance frequencies at some specific angles in single layer and biased bilayer, shown in Fig. 2(g,h), as we previously discussed. Hence a static magnetic field applied perpendicular to the microwave magnetic field is employed to make sure that all the angular dependence FMR of the films can be detected. The red circles are angular dependent dynamic effective fields of different films measured with *H*_0_ = 135 Oe, while the dark line is the fitting curve using Eq. 1. The fitted values of dynamic anisotropies are listed in Table 1.

**Table 1:** Dynamic anisotropies of the films.

Sample	${{\boldsymbol{H}}}_{{\boldsymbol{e}}}^{{\boldsymbol{dyn}}}$ (Oe)	${{\boldsymbol{H}}}_{{\boldsymbol{k}}}^{{\boldsymbol{dyn}}}$ (Oe)	${{\boldsymbol{H}}}_{{\boldsymbol{r}}}^{{\boldsymbol{dyn}}}$ (Oe)
Single FM	0	2.8	0.3
Biased AFM/FM	4.3	2.1	6.1
Unbiased AFM/FM	1.1	2.6	5.5

According to Stiles and McMichael’s model^25^, the direction of rotatable anisotropy depends on the history of the magnetization of magnetic layer. The angular dependence of dynamic properties of the films should be characterized synthetically by uniaxial anisotropy, exchange bias effect and rotatable anisotropy. In the biased AFM/FM, the comprehensive effect of the uniaxial (${H}_{{k}}^{{dyn}}$) and unidirectional (${H}_{{e}}^{{dyn}}$) anisotropy plays greater role than rotatable anisotropy (*H*_*r*_^*dyn*^), i.e. ${H}_{{k}}^{{dyn}}$ + ${H}_{{e}}^{{dyn}}$ > *H*_*r*_^*dyn*^. Hence the magnetizations align with the direction of exchange bias and uniaxial anisotropy in the absence of applied magnetic field. However, in the unbiased AFM/FM, the rotatable anisotropy is larger than exchange bias, while the relation becomes ${H}_{{k}}^{{dyn}}$ + ${H}_{{e}}^{{dyn}}$ < *H*_*r*_^*dyn*^, which means the rotatable anisotropy is predominant in the system, as shown in Table 1. In this case, other anisotropies are not large enough to rotate the magnetization out of the direction of the rotatable anisotropy, the magnetization should lie in the same direction with the rotatable anisotropy. The conclusion drawn from above discussion is that omnidirectional zero-field FMR can be detected in the unbiased AFM/FM, and yet in the biased AFM/FM, the similar results cannot be obtained. Comparing to that of bilayers, the rotatable anisotropy in FM single layer is quite small, thus the single Fe_20_Ni_80_ layer characterize the typical uniaxial anisotropy. Note that, for all of the three samples, there are some differences between static magnetic properties obtained via VSM and dynamical magnetic properties measured by VNA, as for the difference between ${H}_{{e}}^{{sta}}$ and ${H}_{{e}}^{{dyn}}$. It can be considered to be attributable to asymmetric effects in hysteresis loops together with rotatable anisotropy contributions, as shown in other’s works^21, 22^.

**Figure 3:**
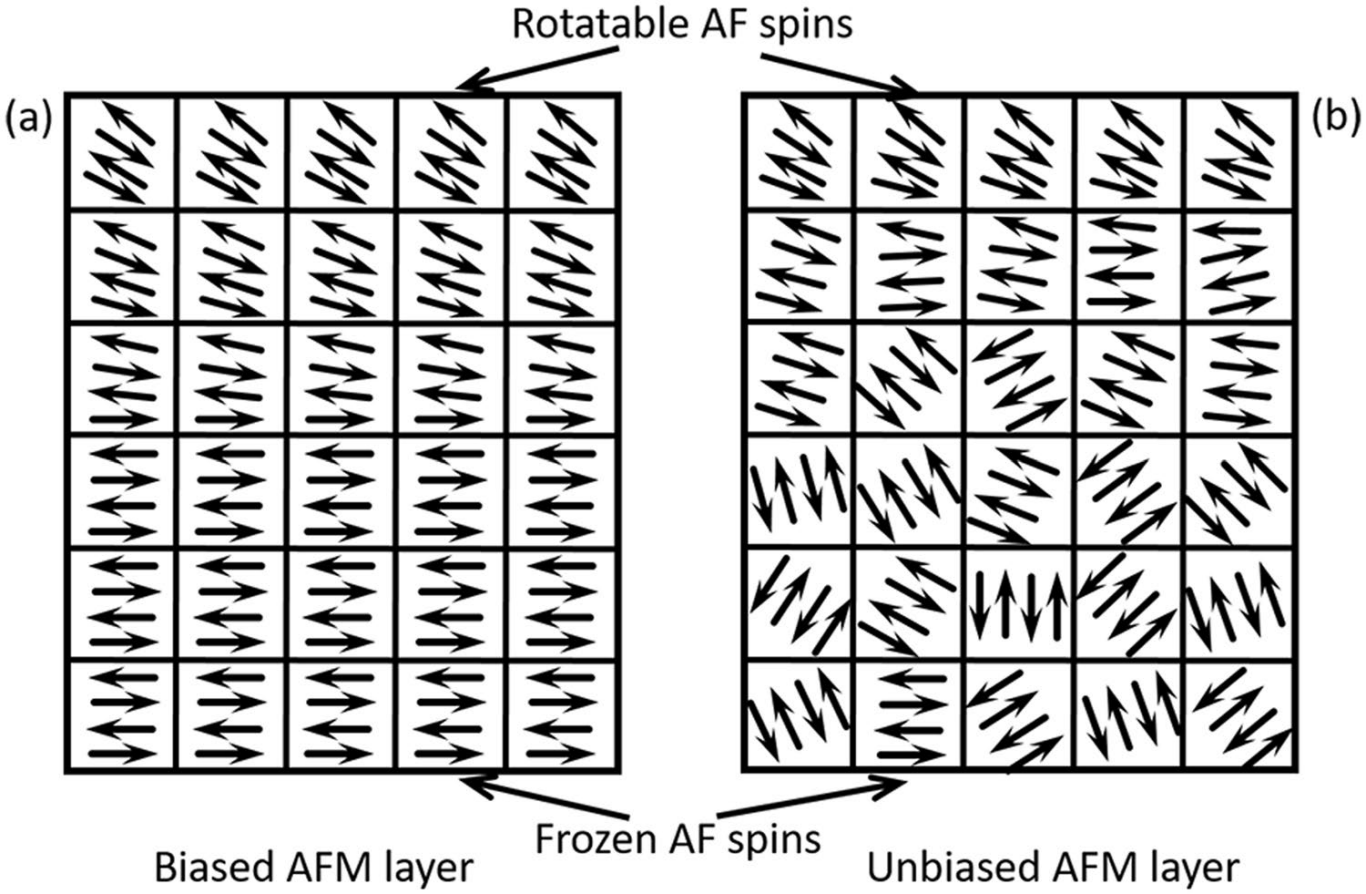
Schematic shows of the spin configurations of AFM sublayer in biased and unbiased bilayer. (**a**) AFM spins in biased AFM/FM bilayer and (**b**) AFM spins in unbiased AFM/FM bilayer. The arrows encased in the squares represent the directions of spins.

The sketch of experimental geometries of AFM spins in both biased and unbiased bilayers are presented in Fig. 3. As discussed above, the spins in AFM of biased AFM/FM aligned uniformly, therefore, an obviously loop shift along EA was observed. While magnetic domains formed in the in AFM of unbiased AFM/FM [Fig. 3(b)]. In such AFM, moments inside the domains are antiferromagnetic, but the domains aligned randomly because of the absence of the external magnetic field during the sample preparation. The slight loop shift along EA was observed in Fig. 1(b) is due to the random pinning of the AFM spins. However, the rotatable spins in AFM layer, which rotates irreversibly as the FM moments rotate, do not depend on the alignment of the spins. Rotatable anisotropy can be resulted in both the biased and unbiased bilayer due to the exchange coupling between the rotatable AFM spins and the FM spins^25^.

**Figure 4:**
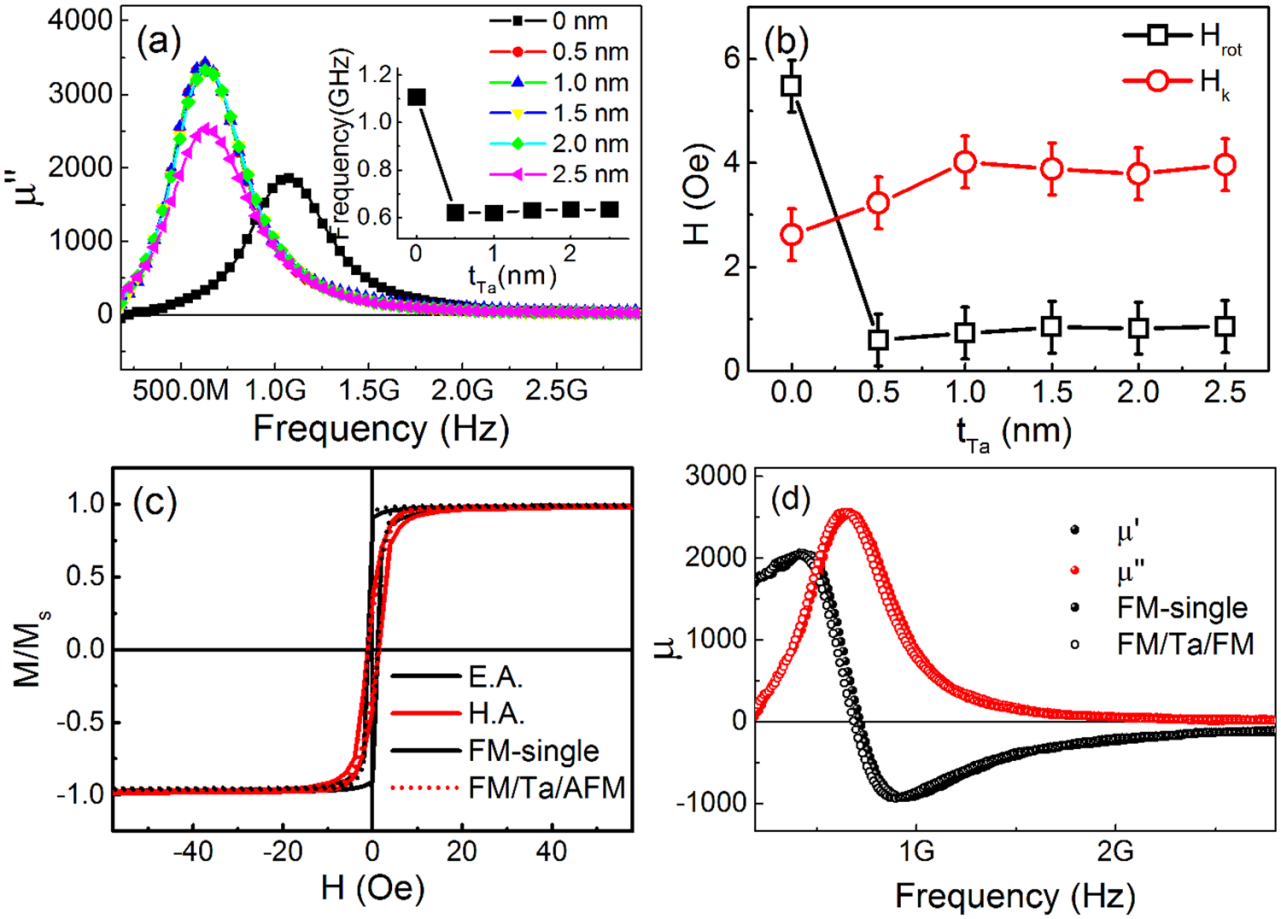
Microwave characteristics of Fe_20_Ni_80_/Ta (x nm)/Fe_50_Mn_50_ bilayers. (**a**) Imaginary permeability of Fe_20_Ni_80_/Ta (x nm)/Fe_50_Mn_50_ along EA with interlayer thickness of Ta (*t*_Ta_) ranges from 0 nm–2.5 nm, the insert shows the variation of resonance frequencies as a function of *t*_Ta_, (**b**) thickness dependences of ${H}_{{r}}^{{\rm{dyn}}}$ (black open square) and ${H}_{{k}}^{{dyn}}$ (red circle), (**c**) and (**d**) are the hysteresis loops and zero-field permeability of the Fe_20_Ni_80_ single layer and Fe_20_Ni_80_/Ta (2 nm)/Fe_50_Mn_50_ multilayer, respectively.

In order to regulate the rotatable anisotropy in AFM-FM systems by controlling the intensity of exchange coupling between AFM and FM layer, a set of samples with increasing thickness of tantalum (Ta) as interlayer was prepared, no external magnetic field was used during the whole deposition. The magnetic properties of the films were performed as shown in Fig. 4. In each of these samples, the Ta interlayer was used to separate the AFM and FM layer. Figure 4(a) shows that the resonance frequencies of the films split by Ta are apparently lower than unseparated sample (*t*_*Ta*_ = 0), the insert of Fig. 4(a) shows exactly the variation of resonance frequencies with increasing *t*_Ta_. The plunge of resonance frequency from 1.1 GHz to 0.6 GHz was observed as *t*_Ta_ increased from 0 nm to 0.5 nm, however, when *t*_Ta_ increase from 0.5 nm to 2.5 nm, the resonance frequencies almost remain the same, which means the direct coupling between FM and AFM spins are separated when *t*_Ta_ > 0.5 nm. The ${H}_{{r}}^{{dyn}}$ as a function of *t*_Ta_ is plotted in the Fig. 4(b), the rotatable anisotropy shows the same tendency of plunging from 5.5 Oe to 0.6 Oe as resonance frequency does. This implies that the rotatable anisotropy driven by exchange coupling between the AFM rotatable spins and the FM spin only existed in the interface of the FM and AFM, and the coupling disappears when an ultra-thin buttering of Ta layer is inserted. Figure 4(c,d) present the comparisons of the magnetic properties of tantalum separated AFM/FM bilayer to those of FeNi single film. We can see that both hysteresis loops and permeability spectra of the bilayer and the single layer are almost coincident, indicate that the exchange coupling of AFM and FM are completely separated by Ta buffer layer, i.e., the Ta separated AFM/FM bilayer shows the same static and dynamic magnetic properties as the FeNi single layer.

In summary, the rotatable anisotropy of about 5 or 6 Oe was derived from dynamic angle dependent measurements of permeability spectra for both biased AFM/FM and unbiased AFM/FM. Particularly for the unbiased AFM/FM, omnidirectional zero-field FMR was measured because of the absence of exchange bias field. This indicates that the rotatable anisotropy in AFM-FM bilayer derive from the irreversible rotates of grains in antiferromagnetic layer while the magnetization in ferromagnetic layer rotates, and it does not depend on the existence of exchange bias effect. In addition, results of a set of Fe_20_Ni_80_/Fe_50_Mn_50_ films with Ta as interlayer illustrates that the rotatable anisotropy can only exist with the exchange coupling between the AFM rotatable spins and the FM spin in the interface of the FM and AFM. The analyzing results and the discussion in this work about the dynamic and static magnetic properties of Fe_50_Mn_50_-Fe_20_Ni_80_ bilayers have further implications in understanding the origin of rotatable anisotropy in exchange biased antiferromagnetic and ferromagnetic films, as well as in various magnetic devices.

## Methods

### Sample fabrication

A radio frequency (RF) magnetron sputtering system was employed for depositing the films. The sputtering chamber was pumped to about 4.8 × 10^−5^ Pa as base pressure, while the chamber pressure was maintained at 0.3 Pa at room temperature during depositing with Ar flow rate around 20 SCCM. The RF power of the 3 inch targets is 50 W, to obtain a felicitous sputtering rate. Three kinds of samples were prepared. By controlling the depositing time, 50 nm thick Fe_20_Ni_80_ as the under layer and 30 nm thick Fe_50_Mn_50_ as the upper layer were deposited respectively onto a 5 × 10 × 0.4 mm Si (111) substrate. Single Fe_20_Ni_80_ layer with thickness of 50 nm and another FeNi/FeMn bilayer with a permanent magnetic field about 240 Oe adjacent to the substrate are deposited as comparison. In addition, another five FeNi/Ta/FeMn trilayers with increasing thickness of Ta (0.5 nm, 1.0 nm, 1.5 nm, 2.0 nm and 2.5 nm) interlayer were deposited to tune the exchange coupling between FM and AFM layers. All of the samples were cut to squares with size of 5 × 5 mm to carry out measurement.

### Measurement

Vibrating sample magnetometer (VSM) and vector network analyzer (VNA) have been employed to obtain the static and dynamic magnetic properties respectively. Hysteresis loops measured by VSM were used to determine both the static in-plane uniaxial anisotropy and the saturation magnetization *M*_*s*_ of each sample. The dynamical response of the films has been characterized by VNA, to obtain the permeability spectrum as a function of external applied field *H*_0_ and the rotating angles that relative to the *H*_0_. Furthermore, the rotatable anisotropy and exchange bias field has been obtained via fitting the angular dependence FMR frequency data.

## References

[1] Fetisov YK, Srinivasan G (2005). Electrically tunable ferrite-ferroelectric microwave delay lines. Appl. Phys. Lett..

[2] Essin AM, Turner AM, Moore JE, Vanderbilt D (2010). Orbital magnetoelectric coupling in band insulators. Phys. Rev. B.

[3] Tatarenko AS, Srinivasan G, Bichurin MI (2006). Magnetoelectric microwave phase shifter. Appl. Phys. Lett..

[4] Song YY, Sun Y, Lu L, Bevivino J, Wu M (2010). Self-biased planar millimeter wave notch filters based on magnetostatic wave excitation in barium hexagonal ferrite thin films. Appl. Phys. Lett..

[5] Zhou Y, Liu Z, Chen Y (2010). Design of a novel tunable microstrip band pass filter. J. Electron. Meas. Instrum..

[6] Che RC, Peng L-M, Duan XF, Chen Q, Liang XL (2004). Microwave absorption enhancement and complex permittivity and permeability of Fe encapsulated within carbon nanotubes. Adv. Mater..

[7] Du H (2015). Self-bias ferromagnetic resonance and quasi-magnetic isotropy of (FeCoB/MgO)_6_ multilayers prepared by composition gradient sputtering. IEEE Trans. Magn..

[8] Prosen RJ, Holmen JO, Gran BE (1961). Rotatable anisotropy in thin Permalloy films. J. Appl. Phys..

[9] Li X, Sun X, Wang J, Liu Q (2015). Magnetic properties of Permalloy films with different thicknesses deposited onto obliquely sputtered Cu underlayers. J. Magn. Magn. Mater..

[10] Zhou C, Jiang C, Zhao Z (2015). Enhancement of rotatable anisotropy in ferrite doped FeNi thin film with oblique sputtering. J. Phys. D. Appl. Phys..

[11] Fujiwara H, Sugita Y, Saito N (1964). Mechanism of rotatable anisotropy in thin magnetic films of Ni, Fe, and Ni-Fe. Appl. Phys. Lett..

[12] Tacchi S (2014). Rotatable magnetic anisotropy in a Fe 0.8 Ga 0.2 thin film with stripe domains: Dynamics versus statics. Phys. Rev. B.

[13] Neudert A, McCord J, Schäfer R, Schultz L (2004). Dynamic anisotropy in amorphous CoZrTa films. J. Appl. Phys..

[14] Suran G (1994). Variations thin films of the local anisotropy. J. Appl. Phys..

[15] Rantschler JO, Alexander C (2003). Ripple field effect on high-frequency measurements of FeTiN films. J. Appl. Phys..

[16] Hoffman H (1968). Theory of magnetization ripple in ferromagnetic films. *IEEE Trans*. Magnetics.

[17] Chai G, Phuoc NN, Ong CK (2012). Exchange coupling driven omnidirectional rotatable anisotropy in ferrite doped CoFe thin film. Sci. Rep..

[18] Myagkov VG (2015). High rotatable magnetic anisotropy in epitaxial L10CoPt(111) thin films. JETP Lett..

[19] Schafer D (2015). Antiparallel interface coupling evidenced by negative rotatable anisotropy in IrMn/NiFe bilayers. J. Appl. Phys..

[20] Blachowicz T (2007). Rotatable anisotropy in epitaxial exchange-biased materials revealed by Brillouin light scattering. J. Appl. Phys..

[21] McCord J, Mattheis R, Elefant D (2004). Dynamic magnetic anisotropy at the onset of exchange bias: The NiFe/IrMn ferromagnet/antiferromagnet system. Phys. Rev. B.

[22] McMichael R, Stiles M, Chen P, Egelhoff W (1998). Ferromagnetic resonance studies of NiO-coupled thin films of Ni_80_Fe_20_. Phys. Rev. B.

[23] Blachowicz T (2007). Exchange bias in epitaxial CoO Co bilayers with different crystallographic symmetries. Phys. Rev. B.

[24] Geshev J, Pereira L, Schmidt J (2002). Rotatable anisotropy and coercivity in exchange-bias bilayers. Phys. Rev. B.

[25] Stiles M, McMichael R (1999). Model for exchange bias in polycrystalline ferromagnet-antiferromagnet bilayers. Phys. Rev. B.

[26] Li Z, Zhang S (2000). Coercive mechanisms in ferromagnetic-antiferromagnetic bilayers. Phys. Rev. B.

[27] Leighton C, Nogués J, Jönsson-Åkerman B, Schuller I (2000). Coercivity enhancement in exchange biased systems driven by interfacial magnetic frustration. Phys. Rev. Lett..

[28] Pan L, Wang F, Wang W, Chai G, Xue D (2016). In-plane isotropic microwave performance of CoZr trilayer in GHz range. Sci. Rep..

[29] Meiklejohn WH (1962). Exchange anisotropy—a Review. J. Appl. Phys..

[30] Berkowitz AE, Takano K (1999). Exchange anisotropy - a review. J. Magn. Magn. Mater..

[31] Kiwi M (2001). Exchange bias theory. J. Magn. Magn. Mater..

[32] Kittel C (1948). On the theory of ferromagnetic resonance absorption. Phys. Rev..

